# Factors Influencing Prescribers' Decision for Extending Venous Thromboembolism Prophylaxis in the Medical Patient Population following Hospitalization

**DOI:** 10.1055/s-0040-1716720

**Published:** 2020-09-11

**Authors:** Alex M. Ebied, Jeremiah Jessee, Yiqing Chen, Jason Konopack, Nila Radhakrishnan, Christina E. DeRemer

**Affiliations:** 1 Department of Clinical Sciences, High Point University Fred Wilson School of Pharmacy, High Point, North Carolina, United States; 2 Department of Pharmacy, Novant Health Forsyth Medical Center, Winston-Salem, North Carolina, United States; 3 Department of Pharmacotherapy and Translational Research, University of Florida College of Pharmacy, Gainesville, Florida, United States; 4 Department of Community Health and Family Medicine, University of Florida College of Medicine, Gainesville, Florida, United States; 5 Department of Medicine, University of Florida College of Medicine, Gainesville, Florida, United States

**Keywords:** VTE prophylaxis, venous thromboembolism prophylaxis, physician influences

## Abstract

**Introduction** Venous thromboembolism (VTE) prophylaxis during hospitalization has clearly defined metrics for risk stratification and practice policy employed to ensure processes of adherence. However, acceptance for practice or even the level and timeline of risk is less clear during the immediate time after hospitalization. With emerging new oral anticoagulant agents, data are available that may influence prescribing in the outpatient setting following hospitalization. A survey was created to determine the level of acceptance or influences for practice surrounding continuation of anticoagulation following hospitalization.

**Methods** This study was designed as a single-center survey of hospitalist and family medicine physician to assess influences to the physician's impression for risk of VTE prophylaxis and knowledge of therapy options.

**Results** Physicians reported depending heavily on medical center protocols for determining anticoagulation at hospital discharge. Prescribing postdischarge anticoagulation was reported to be affected by lack of comfort with prescribing oral medications and concerns with risk of bleeding for all types of anticoagulation outweighing the perceived benefit. Additionally, the decision whether to prescribe these medications at discharge was reported to be related to perceived cost and other patient barriers such as concerns over route of administration.

**Conclusion** Concerns for bleeding were an influence and likely resulted in shorter duration for VTE prophylaxis being prescribed posthospitalization.

## Introduction

The risk for development of a venous thromboembolism (VTE) posthospitalization is increased at time of discharge. 1 Specific patient characteristics can further influence this risk for VTE occurrence which can last for up to 110 days postdischarge. 1 Historically, the assessment of hospitalized medically ill patients for prolonged risk of VTE postdischarge was dependent on the International Medical Prevention Registry on Venous Thromboembolism (IMPROVE) VTE risk assessment model (RAM) and physicians' clinical judgement. 2 Another commonly used tool is the Padua prediction score. 3 Previous clinical trials have assessed the validity of prolonging VTE prophylaxis posthospital discharge and compared current available therapeutic agents for efficacy. 4 5 6 7

As hospitalization is the highest ranked risk factor for the development of VTE, use of VTE prophylaxis for acutely ill medical patients has become a standard of care. 8 Standard prophylaxis is given during hospitalization based on certain risk characteristics, such as a current diagnosis of pneumonia, acute respiratory failure, heart failure, or history of stroke among other criteria associated with increased VTE risk in the acute medically ill population. 9 10 11 The clinical scenarios which require extension of VTE prophylaxis beyond hospitalization are less defined. While the duration has previously been defined as 6 to 14 days posthospitalization, the risk of VTE events remain the highest for the first 30 to 45 days postdischarge. 12 13 With the exception of recommendations to continue posthospitalization VTE prophylaxis for acutely immobilized patients up to 21 days, current guidelines do not offer a standard consensus on duration of posthospitalization prophylaxis or describe the patient population that may benefit from the extended prophylaxis. 14

Direct oral anticoagulants (DOACs) have been of interest in recent studies due to ease of administration compared with enoxaparin for VTE prophylaxis. Current data show both apixaban and rivaroxaban are noninferior to traditional enoxaparin for short durations (10–14 days) postdischarge but notably showed no benefit for a prolonged duration (30–35 days) and increased the incidence of bleeding. 4 5 6 7 An additional clinical trial looked at the efficacy of a new comer, betrixaban, for reducing the risk of VTE postdischarge in a subset of patients considered to be at high risk for VTE prophylaxis. 15 Investigators found benefit in patients with elevated D-dimer or aged over 75 years and in the younger patients with specific risk factors such as a history of VTE or a history of cancer. 7 15 16

Currently, at our hospital, VTE prophylaxis is required for all patients at intermediate or high risk for VTE during the hospitalization. All admission order sets have prompts to encourage either the ordering of VTE prophylaxis or a reason that VTE prophylaxis is not needed. There is currently no protocol in the hospital for VTE prophylaxis postdischarge.

This study is aimed to assess the physician interpretation of data for prolonged VTE prophylaxis posthospital discharge and the factors that may influence physician willingness to prescribe anticoagulants for posthospitalization VTE risk.

## Methods

This was a single-center, anonymous survey distributed to a large academic medical center. At our institution, inpatient admission protocols for VTE prophylaxis contain options for unfractionated heparin 5,000-unit thrice daily or enoxaparin 40-mg once daily, but there is not a protocol for discharge VTE. The 10-question survey was distributed to physicians, who practice in an inpatient setting, with disclosure and intent to assess their interpretation of clinical data for prolonged VTE prophylaxis and investigate influences on practice. All collected survey data were deidentified at the time of collection. Leadership in the Department of Community Health and Family Medicine and the Division of Hospital Medicine supported the distribution of the survey. The survey was distributed through the university Qualtrics platform through e-mail which allowed anonymous responses from participants. All surveys were completed electronically. All physicians were asked to complete the survey between July 2018 and December 2018.

### Statistical Analysis

All the survey responses were reported as percentage frequency. The association between categorical variables and assessment of bleeding or thrombosis risk were evaluated using Fisher's exact test. All statistical analysis was done using SAS version 9.4 (SAS Institute Inc., Cary, North Carolina, United States).

## Results

### Baseline Characteristics

A total of 23 physicians responded to the survey during the study period. The survey was distributed to 40 hospitalist and 51 family medicine providers with a 25% survey response rate. Majority of respondents also practiced in the outpatient setting, with <10 years of experience, while serving as attendings at their practice site ( Table 1 ). Notably, physicians with a primary assignment in the outpatient setting still do work in the inpatient setting for part of the year (4–12 weeks, for example).

**Table 1:** Physician characteristics

Question and answer	Frequency ( *n* = 23)	Percentage (%)
Hospitalist?		
No	20	86.96
Yes	3	13.04
Attending physician?		
No	3	13.04
Yes	20	86.96
Medical resident year		
PGY1	1	8.70
PGY3	1	8.70
Not applicable	21	82.60
Years in practice		
5–10 years	7	30.43
≤5 years	7	30.43
≥10 years	6	26.09
Missing information	3	13.05
Focus area of practice		
Inpatient	3	13.04
Outpatient	20	86.96

Abbreviation: PGY, postgraduate year.

### Questionnaire Response

A summary for the recorded responses to the questionnaire is available in Table 2 . Of the 23 physicians to respond, 19 (82.60%) utilized the medical center's protocol and clinical judgement to determine if patients needed postdischarge VTE prophylaxis. The common deciding factors that would influence prescribing of pharmacologic anticoagulation prophylaxis at discharge were both previous VTE or superficial thrombosis and active cancer (25.86%), limited or reduced mobility (15.52%), and intensive care unit stay during hospitalization (13.79%). The association of increased risk for VTE postdischarge was found for individuals less than 14 days from hospitalization (65.22%) and considered the highest risk in medically ill patients with an active cancer diagnosis (28.57%), previous VTE or superficial thrombosis (23.21%), inherited or acquired thrombophilia (16.07%), or limited/reduced mobility (16.07%). A large portion of physicians (47.83%) agreed the availability of oral pharmacologic agents for VTE prophylaxis would influence their prescribing practices, while the remainder were either impartial to the route of administration or stated it made no difference to their prescribing, 26.09 versus 13.04%, respectively. Only six physicians (26.09%) were aware of betrixaban being Food and Drug Administration (FDA) approved for VTE prophylaxis in medically ill patients for up to 45 days postdischarge.

**Table 2:** Questionnaire and frequency of response

Question	Frequency ( *n* = 23)	Percentage (%)
2. During VTE prophylaxis prescribing during a medically ill patient admitted to the hospital, which of the below influences your selection most		
Medical center has a protocol for all patients	10	43.48
IMPROVE risk assessment model	2	8.70
Clinical judgment	9	39.12
Missing Information	2	8.70
3. Which of the following risk factors for VTE development do you perceive as carrying the highest risk for VTE development and would therefore use pharmacotherapy for prophylaxis? (select top 3)		
Age	2	3.45
D-dimer ≥ 2 × ULN	1	1.72
Intensive care unit stay during hospitalization	8	13.79
Active cancer	15	25.86
Hospitalization ≥ 3 days	1	1.72
Inherited or acquired thrombophilia	4	6.90
Limited/reduced mobility	9	15.52
Lower limb paralysis	1	1.72
Previous VTE or superficial thrombosis	15	25.86
Stroke history	2	3.45
Missing information	3	N/A
4. In your opinion, VTE risk following hospitalization only requires pharmacologic management for what duration?		
> 30 days	3	13.04
≥14 days	2	8.70
< 7 days	11	47.82
≥7–14 days	4	17.40
Missing information	3	13.04
5. Are there particular risk factors, excluding surgical/orthopedic patients, that would influence you to continue VTE prophylaxis following hospitalization (select top 3)		
Age	1	1.79
D-dimer ≥ 2 × ULN	3	5.36
Intensive care unit stay during hospitalization	1	1.79
Active cancer	16	28.57
Hospitalization ≥3 days	1	1.79
Inherited or acquired thrombophilia	9	16.07
Limited/reduced mobility	9	16.07
Lower limb paralysis	2	3.57
Previous VTE or superficial thrombosis	13	23.21
Stroke history	1	1.79
Information missing	3	N/A
6. Would having an oral pharmacologic agent influence you to prescribe a VTE prophylaxis more often?		
1 (Oral makes no difference)	3	13.04
2 (Neutral)	6	26.09
3	6	26.09
4 (Oral makes a complete difference)	5	21.74
Missing information	3	13.04
7. Which of the below choices is a higher concern when considering prescribing or not prescribing a VTE prophylaxis agent following hospitalization?		
Bleeding	11	47.83
Thrombosis	9	39.13
Missing information	3	13.04
8. Are you aware there is a new oral agent FDA approved for VTE prophylaxis in medically ill patient population for up to 45 days following hospitalization?		
No	14	60.87
Yes	6	26.09
Missing information	3	13.04
9. I am likely or not likely to discharge a medically ill hospitalized patient (without surgery) home with VTE prophylaxis because		
Would not prescribe: based on literature, this is NOT a necessary practice	2	8.70
Would prescribe: based on literature, this is a necessary practice	4	17.40
Would prescribe: based on clinical opinion and decision, this is a necessary practice	6	26.09
Would not prescribe: based on clinical opinion and decision, this is not a necessary practice	6	26.09
Missing information	5	21.72
10. In my opinion, regardless of if I prescribe a VTE prophylaxis agent, barriers that will decrease patient adherence (place in order with first being most likely to last being less likely)		
Fear of bleeding		
1	2	11.11
2	1	5.56
3	8	44.44
4	7	38.89
Missing information	5	N/A
Feels unnecessary or will not understand indication		
1	1	5.56
2	5	27.78
3	8	44.44
4	4	22.22
Missing information	5	N/A
Cost barriers		
1	14	77.78
2	1	5.56
3	2	11.11
4	1	5.56
Missing information	5	N/A
Route of medication barriers		
1	1	5.56
2	11	61.11
4	6	33.33
Missing information	5	N/A
Other		
5	18	100.00
Missing information	5	N/A

Abbreviations: FDA, Food and Drug Administration; IMPROVE, International Medical Prevention Registry on Venous Thromboembolism; N/A, not available; UNL, upper limit of normal; VTE, venous thromboembolism.

Physicians were divided on prescribing habits whether they would add pharmacologic VTE prophylaxis postdischarge. Four physicians stated they would have based on current literature (17.40%) or based on current clinical decision making and opinion (26.09%) while eight physicians would not have based on current literature (8.70%) or based on clinical decision making and opinion (26.09%). When asked what raises a higher concern for prescribing or not prescribing VTE prophylaxis, a majority of physicians consider bleeding (47.83%) as a worse outcome than potential thrombosis (39.13%).

When asked to rank their considerations for barriers that decrease patient adherence regardless of prescribing practices, a majority (77.78%) ranked cost as being the most influential followed by route of medication (61.11%), and the patient feeling VTE prophylaxis is unnecessary or will not understand the indication for use (44.44%).

### Outcomes

We evaluated the relationship between response to questions 2, 7, and 8 ( Table 2 ). Physicians who responded the highest risk for VTE posthospitalization was <14 days postdischarge in turn utilized the medical center protocol and their clinical judgement for prescribing VTE prophylaxis postdischarge (95.45%; *p* = 0.008). The association between the use of the medical center protocol and clinical judgement also correlated with their concern of bleeding postdischarge (55%; *p* = 0.07), prescribing or omitting postdischarge VTE prophylaxis–based clinical judgment or current literature ( *p* = 0.0015), and physicians who associated with a previous VTE or superficial thrombosis, active cancer diagnosis, or limited/reduced mobility (67.24%; *p* < 0.001). The knowledge of a new pharmacologic agent for postdischarge VTE prophylaxis did not correlate with a difference in prescribing practices ( *p* = 0.13).

Physicians who responded bleeding was a higher concern postdischarge than the potential for thrombosis also associated the highest risk for VTE prophylaxis posthospitalization was <14 days ( *p* = 0.0118). They also responded active cancer, previous VTE or superficial thrombosis, and limited/reduced mobility influenced their prescribing habits ( *p* < 0.0001), and for the same factors when assessing the need to continue VTE prophylaxis postdischarge ( *p* = 0.0003). Their consideration of clinical judgment or current literature for prescribing or omitting VTE prophylaxis postdischarge was not associated with their concern for bleeding or thrombosis ( *p* = 0.0555).

The knowledge of a new pharmacologic agent indicated for VTE prophylaxis after hospitalization for up to 45 days also correlated with prescriber assessment of the highest risk for VTE is <14 days after discharge ( *p* = 0.0114). The association of active cancer, limited or reduced mobility, and previous VTE or superficial thrombosis was also associated with knowledge of publications relating to betrixaban for VTE prophylaxis ( *p* = 0.0004) and consideration for continuing VTE prophylaxis postdischarge ( *p* < 0.0001). The assessment of bleeding versus thrombus postdischarge for prescribing VTE prophylaxis was not associated with the betrixaban literature ( *p* = 0.1073).

## Discussion

Clinical judgement and risk assessment for VTE prophylaxis play a critical role in reducing the risk of VTE postdischarge in medically ill patients. 17 18 19 20 Newer data have not shown benefit for the extended duration of VTE prophylaxis in the general population of medically ill patients post–hospital discharge. 4 5 6 The targeted utilization of extended VTE prophylaxis in a subset of patients, including those with elevated D-dimer and those over the age of 75 years with an elevated D-dimer may be indicated as those specific populations may benefit from extended VTE prophylaxis up to 45 days of postdischarge. 17 That stated, D-dimer elevations associated with medical conditions not requiring anticoagulation are commonplace in the opinion of the authors.

The assessment of physician perspective and assessment of clinical literature provides a new perspective on application of current clinical trials. The questionnaire provided insight into what providers consider applicable to their practice and what is taken into consideration when prescribing pharmacologic agents for VTE prophylaxis.

A majority of physicians utilized clinical judgement and patient risk factors (e.g., lack of mobility, active cancer, and previous VTE) to assess the need for prescribing pharmacologic agents to prevent VTE after discharge. Application of the IMPROVE VTE risk score did not play a critical role in the physician prescribing habits or their assessment of VTE risk. The association of outcomes between pharmacologic agents was not taken into consideration and the use of oral versus injectable products did not play a role in prescribing habits. A shorter duration (e.g., 7–14 days) for medically ill patients postdischarge was preferred by physicians despite evidence for extended intervals of therapy in high-risk patients. 6

The increased risk of bleeding described in previous trials may be associated with the limited use of extended VTE prophylaxis in medically ill patients. 4 5 In addition, when assessing patients for continuation of VTE prophylaxis postdischarge, physicians indicated that factors, such as the cost of oral agents, route of administration, and patient understanding of indication, play a critical role in prescribing habits. This supports physician prescribing habits based on both risk of bleeding and practicality for utilization in an individualized patient approach. This sentiment was supported further by expert commentary that approximately 90% of physicians do not use prophylaxis for medically ill patients at time of discharge. The main cited reason was related to the concern of bleeding outweighing clinical benefit and ongoing acute medical illnesses. 21 Prescribing habits of physicians completing the survey were associated with their clinical judgement instead of current clinical data. Future studies are needed to assess factors that influence prescriber's decisions on the use of extended VTE prophylaxis in medically ill patients.

The ACCEPT Study was conducted in Germany as an observational, retrospective design, and included a surgical and medical population while excluding patients with prior thromboembolism. Overall, 64.9% of medical patients were recommended to have continued VTE prophylaxis which was implemented in 94.3% of those evaluated by office-based physicians. 22 This study offers an alternative success rate for continuing VTE prophylaxis, but when coupled with attitudes for deprescribing at time of discharge to minimize polypharmacy, extended VTE prophylaxis will continue to face barriers to implementation despite continued educational efforts. 23

## Limitations

First, the survey utilized in this study is a novel assessment tool and requires further evaluation for validity of clinical use to assess prescribing habits of physicians for postdischarge VTE prophylaxis. Second, the assessment of individual patient factors (e.g., additional diagnoses, clinical perspective, and multiple hospitalizations) were not taken into account when completing the questionnaire. Third, the low-inclusion rate of physicians may limit the statistical analysis and association of study data to a larger cohort of physicians within a practice setting. Finally, the data collected was primarily from physicians in the outpatient setting, not the acute care setting. The continuation of VTE prophylaxis postdischarge may differ in physicians that practice primarily in the inpatient setting or split time between the two.

## Conclusion

Current clinical practice standards for physicians assessing the need of extended VTE prophylaxis in medically ill patients after hospitalization are correlated with clinical judgement, assessment of bleeding versus thrombosis risk, and individualized patient risk factors for risk of thrombosis after discharge. The addition of new clinical trials to extend the indication of VTE prophylaxis or availability of oral pharmacologic agents did not play a role in prescribing habits. Independent validation and assessment of the survey for current clinical practices in prescribing VTE prophylaxis should be undertaken to assess the current standard of care for the continuation of VTE prophylaxis postdischarge for medically ill patients. Additionally, further studies to determine whether directed interventions might change physicians' perceptions and practices at the time of hospital discharge may be of benefit.
